# A qualitative analysis of the impact of canine hypoadrenocorticism on the quality of life of owners

**DOI:** 10.1186/s12917-023-03716-y

**Published:** 2023-09-09

**Authors:** Julia Hupfeld, Michael Dölle, Holger A. Volk, Johanna Rieder

**Affiliations:** 1 Department of Small Animal Medicine and Surgery, University of Veterinary Medicine Hannover, Foundation, Hannover, Germany; 2 Department of Gastroenterology, Hepatology and Endocrinology, Hannover Medical School, Hannover, Germany

**Keywords:** Hypoadrenocorticism, Dogs, Owners, Quality of life

## Abstract

**Background:**

Canine hypoadrenocorticism is a rare chronic disease, which demands intense dog-owner interaction, as its treatment requires to be individualised. The aim of this study was a qualitative analysis of the challenges owners face when dealing with the disease, especially regarding its management and how this affects quality of life. By promoting an online discussion between owners, we transcribed and summarised their experiential knowledge in dealing with the disease.

**Methods:**

Owners were recruited for the online seminars via social media. After a theoretical introduction, participants were free to share experiences and ask questions. The recorded events were retrospectively analysed.

**Results:**

Twenty-four owners of 22 Addisonian dogs took part in four events. Owners felt most “traumatised” when experiencing their dog’s acute adrenal crisis. The initial adjustment phase and distinguishing the non-specific symptoms of hypoadrenocorticism from those of other diseases were also challenging. Overall, owners were well informed on the disease and committed to its long-term adjustment.

**Conclusions:**

Adrenal crisis and the initial adjustment phase may be more burdening to owners than expected. Understanding what their clients’ concerns are, can help veterinarians provide better care and reduce the negative impacts of canine hypoadrenocorticism. Promoting peer to peer support, as well as providing a framework for participative communication might also help.

## Introduction

Canine hypoadrenocorticism is a rare immune-mediated [1, 2] adrenal gland insufficiency, with an estimated prevalence of 0,06% [3]. Affected dogs are unable to produce cortisol and aldosterone, vital hormones to adequately face stress-reactions, help with the integrity of gastrointestinal tract, to preserve normoglycemia, as well as water and electrolyte homeostasis [4]. Clinical signs are unspecific [4] and its clinical relevance rests mainly in the possibility of evolving into a life-threatening acute adrenal crisis [5]. This occurs when there is an absolute (before diagnosis) or relative (after diagnosis) deficiency of the aforementioned hormones. Its low prevalence and unspecific manifestation often result in delayed diagnosis [6], which was perceived as frustrating and burdening to owners in a recent study [6]. Its treatment consists of life-long hormonal substitution of glucocorticoids and if needed, mineralocorticoids [7]. Dosing regimen is individually set and demands intense owner-dog interaction, especially regarding the glucocorticoid replacement therapy, which needs to be adjusted according to stress situations [5]. Caring for a sick dog reportedly increases stress and reduces quality of life (QoL) of owners of companion animals with chronic or terminal disease [8]. Since adequate pharmaceutical treatment of pets depends solely on their carer’s adherence [9], it is of great importance to invest in a relationship-centred care [10], enabling shared decision-making [11]. Previously published studies into qualitatively analysing impacts of chronic diseases [12, 13] on the quality of life of owners have made successful in-depth analyses and discovered novel factors. This study is the second phase into analysing QoL of owners and their dogs with hypoadrenocorticism. The first part of the study consisted of a quantitative analysis [6]. In the present study, the aim was a qualitative analysis of the impacts of canine hypoadrenocorticism on the owners’ QoL, as well as gaining more information on the long-term treatment, especially regarding the glucocorticoid replacement therapy. In addition, our study aimed to support patient-owners with the decision-making process regarding the initial and long-term adjustment.

## Materials and methods

Owners were recruited for online meetings during a former questionnaire study [6]. Additionally, clients of the Dept. for Small Animal Medicine and Surgery of the University of Veterinary Medicine Hannover were invited to participate via email. The online events happened in June and July 2021 via Microsoft Teams and were recorded for subsequent analysis. They were semi-structured and held in German language. In the beginning, a short introductory presentation on canine hypoadrenocorticism was given, after which 15 items embracing three main topics were discussed: 1. Onset of disease (e.g., initial clinical signs, age at diagnosis, occurrence of acute adrenal crisis), long-term therapy adjustment (e.g., initial and current medication regimen, boosting regimen, stressors, presence of side effects) and everyday life with an Addisonian dog (e.g., changes after diagnosis, fears regarding the disease). Subsequently, owners were encouraged to freely share their experiences and ask questions. The videoconferences were recorded and transcribed. Relevant sections were translated and are stated in quotation marks in the result section. The qualitative analysis was performed using thematic analysis, a six-step method in which patterns within the data are identified, interpreted, and contextualized [14]. All participants provided informed consent.

## Results

A total of 24 owners of 22 dogs with hypoadrenocorticism participated on the four events we promoted, two of them were previously known clients of our clinic. The mean duration of the events was 1 h 45 min (range, 1h24min-2 h). The interviews occurred on the following dates: June 24th, July 1st, 13th and 22nd of 2021. Five, eight, seven and four patient-owners took part, respectively. Overall, owners were very well informed on canine hypoadrenocorticism, and the events led to an informative exchange. Thematic analysis allowed us to identify key themes, three of which were: (1) emotional impact of canine hypoadrenocorticism on owners, (2) challenges of long-term treatment and (3) changes in everyday life of dogs and their owners.

### Emotional impact of canine hypoadrenocorticism

To most owners, experiencing their dog’s adrenal crisis was emotionally burdening. For many, the burden was amplified by the long time it took for diagnosis to be reached, with owners having to witness the health of their dogs deteriorating rapidly, and veterinary surgeons being unable to determine the cause. Many described that primary care veterinary surgeons struggled to reach the diagnosis of HA. The owners were worried that their dogs would die in the meantime, as a diagnosis was not reached. One owner said: “*The experience of an adrenal crisis was absolutely traumatic! I have to say that the 5 days where Clara really went downhill were terrible, so I really needed months afterwards to recover.*” Another owner continued: “*(…) so of course it blows your mind when they say we don’t know if your dog will survive the night.*” “*Leaving her there and having the feeling that she would die alone - that was very bad for me*.”

When asked what helped in this situation, many gave the same answer: they tried to learn as much as possible about the disease to be able to familiarise themselves with the situation.

*Improving my knowledge gave an incredible amount of security, knowledge is insanely reassuring.*

Finding peer support through social media groups was also of great importance to them.

*I didn’t know what to do and this group really took me by the hand.” For others, however, the amount of information shared on social media was overwhelming and just made them feel more insecure about the disease’s management. “I also signed up for this group on Facebook, read it and thought: Oh God, oh God - that made me feel so insecure.*

Many participants reported that onset of acute adrenal crisis happened right after an extremely stressful situation for their dog, a so-called trigger. For one dog, it was right after he was rescued, got vaccinated and neutered in a very short period. For another, it was a family vacation at the beach. Another owner said that an anaesthesia to perform an ear wash was the trigger for their dog. We also asked if any of the dogs had suffered a repeted acute adrenal crisis after the start of treatment, and apart from one dog, none of them had. The one dog that suffered a second adrenal crisis was still in very early stages of treatment and had comorbidities (heart disease and myasthenia gravis).

### Challenges of the long-term management

Participants also described the phase after diagnosis as challenging. Owners felt worried that they could not interpret their dogs’ clinical signs correctly and act accordingly. They also felt overwhelmed to identify potential stressors and how to deal with these, e.g., giving their dogs a “boost” in treatment accordingly. Then on the other side of an adrenal crisis, they mentioned the difficulty of reducing the dose of the medication, especially after experiencing the traumatic experience of acute adrenal crisis and fearing for their dog’s life.

*You really struggle with the diagnosis, that your dog is still alive, then it gets the vital medication and reducing it or giving less is not so easy.*

*It is a real struggle to find the right balance between protecting the organs and keeping the dog fit and well.*

We asked each participant what stresses their dogs, and they reported long car rides, visits to the veterinarian, thunderstorm, strangers in the house, and heat. Interestingly, one owner that had two dogs with hypoadrenocorticism reported opposite behaviours in response to ambient temperature: one Golden Retriever, which was reportedly stressed by excessive heat and one Chihuahua, which needed a higher dose of prednisolone on cold days. Some owners reported having difficulty in recognizing what stresses their dogs and when to boost them, especially those that had been diagnosed just recently. One of them reported being afraid to increase the dose, due to fearing even more side effects than were already present, specifically increase in liver enzyme activity.

*What stresses him out are family disagreements or when there’s a fight or something - he can’t cope with that at all, and it’s interesting because he always wants to get out of the house.*

When asked how an underdosage of glucocorticoids manifests itself on each dog, many reported faecal consistency getting softer. One owner reported that its dog regurgitates when he appeared stressed, and that this is a very clear sign that he needed to be boosted. Another owner noticed that her dog starts to limp when stressed, and that this is a signal for her to increase the dose of prednisolone. Some of them reported that their dogs got lethargic and that with time, they got a feeling of noticing when their dogs need an additional dose. One owner said that several times they only discovered new stressors because of their dog’s typical signs when being underdosed, after the stressful situation had already happened, and that this was a learning experience to intervene sooner in the future.

*I notice it when he doesn’t calm down, when he can’t relax, I give him something (additional prednisolone dose) and after 20 min he lies there and is like oh God, now I’m okay!*

*You learn from one situation to the next, it’s really like that, you get to know your dog.*

Some owners had an interesting approach to managing stressful situations:

*I deliberately don’t take my dog out of the situation because I think the more situations the dog knows, the more relaxed he becomes.*

An owner of a hound dog breed which is naturally easily scared, reported her method:

*He has to cope with everyday situations and when he gets used to it, it’s no longer so exciting. If I know that it’s going to be really intense and he’s going to run for 3 h, for example, then he’ll get it beforehand.*

We also asked about timing and dose of boosts. When a stressful situation was foreseeable, e.g., planned visits to the veterinarians, long car rides, they boosted their dogs in advance. When a stressful situation occurred without the dogs having received a boost, many said that it varied according to the reaction of the dog. Most owners double the dose (increase of 100%) of glucocorticoids when foreseeing stress for their dogs. One owner reported that their dog needed an additional dose on the day following the stressful event, which was confirmed by other participants. Another participant wanted to know if others had, like her, observed that their dogs needed a daily increased dose of glucocorticoids when getting older. This was not confirmed by others.

*I notice it in his whole behaviour somehow and then I try to add either a quarter or half a milligram tablet, so really in smaller amounts to see how he reacts, if it is sufficient … so far, it has always been sufficient.*

An experienced owner shared that massages, specifically the Tellington TTouch^®^, have helped her calm her dog efficiently and avoided the need of a boost several times.

Many owners described the time in which the mineralocorticoid desoxycorticosterone pivalate (DOCP) came to the market in Germany and how they experienced the switch from fludrocortisone to DOCP, or in other cases, reasons for not performing it. A few owners described a great improvement in QoL and side effect profile of their dogs as a result from the switch. Apart from that, one owner reported to feel safer with the replacement therapy as an injection, since potential gastrointestinal disbalances such as vomiting do not interfere with it.

*“This was the first time in a long time that I had the feeling of having a healthy dog in front of me again.” (*After the switch from fludrocortisone to DOCP).

One owner reported that the switch would not have been beneficial for their dog, because trips to the veterinarian represented an extreme amount of stress to him and he was well-adjusted with his oral medication.

Many owners described the process of finding the right dose of DOCP as exhausting and long. Two described being in the process for over a year and still not having reached the minimal dose necessary. Most of the participants of our study (16/24), reduced the dosage of DOCP according to the potassium concentration exclusively, which means the dog only got injected when its concentration was above the mean of the laboratory’s reference interval. If it was under the mean concentration, the dog did not get injected, and the owners had to return for a follow-up of electrolytes after a week. Every postponed week represented a reduction of 10% on the dose of DOCP, and the maximal reduction performed was of 50% of the dose. One owner described having to return to the veterinarian over a four-week period before being able to inject the depot preparation. Another owner reported also having to wait several weeks and the dog only getting injected when it was already presenting clinical signs of underdosage of mineralocorticoids. Other owners reported that the dosage got adjusted according to the labelling (sodium/potassium ratio). They stated not being able to attend to follow-ups so often and additional costs as reasons for having chosen this method.

Another great aspect approached was the client-veterinarian relationship and how it affected the patient’s treatment as well as the owner’s attitude towards it. Participants described the frustration related to not feeling heard by veterinarian professionals when suggesting or discussing matters concerning their dogs and its treatment. These situations were mostly described when owners self-educated via the internet.

*“I would have liked to have had a little more support from my vet.”* Owners that had been through different experiences considered themselves lucky: *“I have to say I’m really lucky that my vet listens to me and we’re in this together.”*

One owner reported that his primary care veterinarian initially had no experience with hypoadrenocorticism and that after his dog was diagnosed, they sought information and made shared decisions about long-term adjustment. Thereafter, the vet reportedly diagnosed and treated other dogs with adrenal insufficiency. The participant continuedly emphasized how important it was to have a good relationship with the attending veterinarian.

### Changes in everyday life

When asked about changes in everyday life, answers varied according to the time owners had been living with their dog’s adrenal insufficiency. Owners of dogs that had been recently diagnosed described having a hard time adjusting to the changes and constantly being afraid of an acute adrenal crisis reoccurring. Owners of dogs that had been living with hypoadrenocorticism for a long time had a more relaxed attitude about lifestyle changes. Overall, most described a persistent state of concern, of always observing their dogs with worry

*I have the feeling that I’m totally sensitive and I’m always worried that I don’t recognise something or that there’s something I don’t perceive or misinterpret - that’s somehow very difficult for me in our routine.*

Many also described fear of not recognizing another disease, due to the unspecific character of hypoadrenocorticism’s manifestations, and nearly every clinical sign being attributable to it. All of them reported to present their dogs to veterinarian professionals more often than they would a healthy dog, not only because of the long-term management, but also because of the persistent fear of an adrenal crisis. By observing and learning to read their dogs, owners reported to have a much stronger bond with them, than they would have with a healthy dog. Some interpreted this as a positive side effect of adrenal insufficiency

*I can also confirm that we have a much closer bond. He is not my first dog; he is my fourth dog now and it is definitely different.*

Apart from her Addisonian dog, one participant mentioned that she has an epileptic dog that gets phenobarbital. She noticed the potassium concentration of this dog was repeatedly at the upper reference interval and wanted to know if we had already observed a dog developing acute adrenal crisis due to the administration of phenobarbital in the routine of our hospital. Owners that have been living with its dog’s disease for a longer time have an overall relaxed view of the lifestyle changes. They described it as daily routine and gave tips to owners of dogs that had been recently diagnosed

*We have our rituals during the day, where I notice how she is doing. Every evening she gets a slice of rusk and I see how she reacts to it: does she do it like she always does, and does she like to eat it or is she a bit slower than usual? So, these are like cornerstones for me throughout the day, which I can use to determine whether she is doing well or not.*

*At first I was afraid, but after a while I knew that this was part of everyday life, then you realize that the animal is doing well and that’s all that matters.*

*I knew at some point that she would definitely not die from Addison’s, and after 10 years of living with it, she really didn’t die from it.*

We have also asked if the dogs were the same as before the diagnosis and nearly every owner agreed that mostly, they were. Participants described that it might have taken a few months for them to get there, but on the long term, they had a completely normal life.

*“We did man trailing again for the first time the other day and I didn’t have to give him any extra boost, I hadn’t seen my dog so happy in the last few months, as happy as he was that day!”* (a dog that had been diagnosed approximately 6 months prior).

*After 2 months, I slowly started to practice agility again with Polly. She didn’t have any diarrhoea or vomiting, nothing at all. Her coat was a little thinner in between, but now it’s great again and, as I said, I actually have a completely normal dog at home.*

## Discussion

The current study examined a population of owners of dogs with hypoadrenocorticism via online events in the form of videoconferences. It is the first study to do a qualitative analysis of the long-term management and its impact on the quality of life of owners. The aim of this study was a qualitative analysis of the long-term therapy adjustment and hypoadrenocorticism per se on the dogs’ and their owners’ quality of life. By promoting online events for owners of affected dogs to exchange information in a semi-structured way. We also aimed to learn from their knowledge and to reduce the impacts of caring for a sick dog. It has been reported that support group intervention might have a positive effect on psychological well-being, stress, burden, and feelings of isolation [1, 2].

Confirming our quantitative findings [3], reoccurrence of acute adrenal crisis represents a great and constant fear for owners of Addisonian dogs. Affected dogs are mostly young [4], so it is comprehensible that having experienced them fighting for their lives due to chronic disease might be traumatising to owners. This fear might also be the reason why it can be hard for owners to reduce the dose of the medication that is keeping their dogs alive. However, it is long known that systemic glucocorticoids in non-physiological doses cause an extent list of short- and long-term side effects [5, 6]. Apart from that, chronic glucocorticoid use in high doses might also lead to acquired glucocorticoid resistance, due to the downregulation of the glucocorticoid receptor [7], which would represent an additional challenge in the long-term management. To prevent that, a low-dose prednisolone treatment respecting the physiological dose of 0,05 mg/kg-0,2 mg/kg is advisable.

Boosting of glucocorticoid (GC) therapy is also a method that helps lower its daily dose. It consists of increasing the dose of glucocorticoids when a stressful situation is foreseeable or has happened and the dog shows signs of underdosage. This allows the GC dose to be as low as possible on “regular” days. We asked our study participants how underdosage manifests itself and to many, softer faecal consistency was a clear sign. It is known that physiological quantities of GCs have a gastroprotective role, including maintenance of glucose homeostasis, gastric mucosal blood flow, attenuation of enhanced gastric motility, mucus production and microvascular permeability [8]. One owner described regurgitating as a clear sign of acute stress for her dog. A possible explanation to this phenomenon might be the transient development of a megaoesophagus, which has been previously reported in other three dogs with hypoadrenocorticism [9–11]. The cause of this remains unknown, but myopathy due to cortisol-deficiency is discussed. Interestingly, one owner mentioned a massage technique called the Tellington TTouch^®^ as a way of reducing acute stress for her dog. The use of this method has successfully reduced secreted cortisol levels in horses’ saliva, when they got moved to a transportation vehicle [12].

Reasons for boosting are individual to each dog, but many of our study participants mentioned visits to the veterinary clinic and long car rides as reasons for adjusting GC dose. For other dogs, however, car rides did not represent a source of additional stress. Excessive heat was also mentioned by owners, which has a microbiological explanation: heat shock proteins, which are synthetized in response to physical, chemical, or biological stresses, including heat exposure [13], interact with the GC receptor, forming a high affinity steroid-binding conformation [14]. Therefore, glucocorticoids are metabolized faster, which explains the need to increasing them for dogs that have adrenal insufficiency. Interestingly, an owner reported his dog needed a higher GC dose when it was cold. The Chihuahua being a dog breed originating from Mexico, and the fact that it is considered the smallest breed to exist might be reasons that explain its sensitivity to cold. Another possible explanation might be that cold temperatures can reduce tendon elasticity and alter the viscosity of the synovial liquid [15], which means that perhaps the Chihuahua could have been suffering from chronic joint pain. Chronic pain might also be the reason why the daily dose of GC of one dog increased when it got older. In human medicine, osteoarthritis, for example, among other typical age-related diseases [16], is reported to have a relationship with higher cortisol levels [17].

Finding the correct dosing regimen takes time and was perceived as emotionally and financially burdening to owners, but it is of great importance to minimize the side effects of the medication and reduce costs on the long run. As confirmed by our study population, the switch from fludrocortisone to DOCP has led to an improvement of the side effect profile, presumably due to the absence of glucocorticoid action in the preparation [18]. High doses of DOCP have also been associated to side effects [19], and are mostly not necessary, except to young dogs, that are still in their growth phase [20]. Our study participants showed great interest and knowledge in the dosing reduction process and the aspects influencing it, especially those that used the reduction method according to the mean potassium concentration. To our knowledge, the described adjustment method has not been published and is presumably the result of empiric knowledge gathered and shared by owners of Addisonian dogs. It is important to remind that defining sodium and potassium concentration does not represent the only way to determine adequate aldosterone replacement. In human medicine, plasma renin activity, as well as blood pressure are additional indicators of adequate mineralocorticoid substitution [21, 22].

Interestingly, a question of an owner regarding treatment with phenobarbital associated with an acute adrenal crisis has been previously reported [23]. This case report discusses that the administration of phenobarbital might have been the trigger of the acute adrenal crisis, but the dog must already have had borderline hypoadrenocorticism prior to it. One of the discussed reasons is that it may interfere with the adrenal axis by accelerating the metabolism of endogenous steroids, which has also been reported by other studies [24].

There are a few limitations associated with this study. The sample size is small and from one country exclusively, however, participants were from all over Germany, which may have increased the universality of results. Only owners with access to the internet and social media participated, possibly lowering the proportion of underprivileged and elderly participants. Furthermore, as we used mainly Facebook, we could also have not had sufficient representation of the younger generation and a sex bias towards middle-aged females.

Although some owners referred to an increase in the human animal bond as a positive side effect of canine hypoadrenocorticism, their reports of constant worries, for example, verifies research which show an increase of the burden when caring for a sick dog [25]. It is only natural that owners tend to look for peer support through social media, as it provenly reduces emotional burden and feelings of isolation related to caring for a sick individual [26, 27].

It is of great importance, however, that veterinarians keep an open mind about this behaviour. Self-information also reportedly helped our study participants, especially right after diagnosis was set. This highlights the importance of veterinary professionals- clients communication. Interestingly, a recent study showed that veterinarians that welcome owner’s interest in further (self-)information, are more likely to have better veterinarian-animal owner-relationships [28]. Veterinary professionals must invest in the relationship with their clients because it might not only reduce the impacts of receiving such a diagnosis, but also improve adherence to treatment [29]. Our study participants reportedly suffered due to having the feeling of not being heard by attending veterinarians. A validated tool which measures QoL of owners (DOQOL) [30] could aid with the process of better understanding the relationship between owning a dog and QoL.

A way of reducing the subjectivity of the long-term management might be health-related quality of life (HRQoL) tools, which have already been published for Cushing’s disease (CushQoL-pet) for example [31]. The clinical signs of over substitution of prednisolone mimic those of hyperadrenocorticism. Those of over dosage of trilostane might mimic clinical signs of hypoadrenocorticism, therefore the mentioned tool might be applicable to Addisonian dogs. The authors suggest weekly evaluation of the affected dog’s health profile, which include aspects such as drinking and urination behaviour, coat quality, eating habits, weight, general activity, and interaction levels. An important addition to the aforementioned items presented in the CushQoL-pet for Addisonian dogs is faecal consistency, which was reported by many participants as a sign of underdosage of prednisolone. This tool might help owners identify which clinical signs are important when monitoring the long-term management, as well as aid clinicians on the clinical assessment of affected dogs on follow-ups. Furthermore, an emergency card containing information about what to do in case of an apparent underdosage can be tailored to each dog. Based on data of the first phase of our study, owners could be instructed to increase the dose of prednisolone by 100% in case signs of an underdosage manifest. In case the dog needs it for more than 3 days, medication should be tapered off until baseline dose is attained again. This means owners of affected dogs should always have a stock of additional prednisolone to prevent an acute adrenal crisis.

Fear of recurrence of acute adrenal crisis is the source of greatest concern to owners of dogs with hypoadrenocorticism and is presumably also a source of concern to attending veterinarians. The knowledge that recurrence of acute adrenal crisis was not suffered by most of the dogs in our study population serves as reassurance to both. Confirming published studies, hypoadrenocorticism has an excellent outcome [32], for most dogs receiving appropriate replacement therapy, and affected dogs were considered the same as before the diagnosis by the majority of our study participants.

In conclusion, acute adrenal crisis was traumatising to owners, that even years after first diagnosis, its reoccurrence was a constant worry. However, apart from one dog of our study population, none suffered from acute adrenal crisis after treatment initiation. Owners felt the need to be heard by attending veterinarians, so investing in relationship centred care might be the key to providing adequate support and enabling shared decision making. This is of great importance in the initial adjustment phase. Emergency cards as well as using validated QoL measurement tools could also aid owners and clinicians in the long-term therapy adjustment process. Overall, this study confirmed a good to excellent prognosis when managed appropriately. Owners described their dogs to be as healthy and active as prior to the diagnosis when appropriate replacement therapy was given.

## Data Availability

The datasets used and/or analysed during the current study are available from the corresponding author on reasonable request.
